# Anti-Obesity Effects of Onion Extract in Zucker Diabetic Fatty Rats

**DOI:** 10.3390/nu4101518

**Published:** 2012-10-22

**Authors:** Orie Yoshinari, Yoshiaki Shiojima, Kiharu Igarashi

**Affiliations:** 1 Faculty of Agriculture, Yamagata University, Tsuruoka, Yamagata 997-8555, Japan; Email: igarashi@tds1.tr.yamagata-u.ac.jp; 2 Ryusendo Co., Ltd., Toshima-ku, Tokyo 171-0021, Japan; Email: y.shiojima@ryusendo.co.jp

**Keywords:** onion, anti-obesity, type 2 diabetes, Zucker rat, sulfur-containing compound

## Abstract

Anti-obesity effects of onion extract were determined in obesity and diabetes-prone Zucker diabetic fatty rats by measuring the efficacy of markers concerned with diabetes and obesity. Body and adipose tissue weights in 5% of onion extract-fed group were found to be significantly lower than the control group without onion extract. Fasting blood glucose and HOMA-IR levels were also improved, although the serum insulin and leptin levels did not show any remarkable difference. Serum triglyceride and free fatty acid levels in both the 3% and 5%-fed group were found to be reduced compared to the control group. Additionally the feeding of the onion extract increased the glucose tolerance. These results suggest that dietary onion extract is beneficial for improving diabetes by decreasing lipid levels. We also examined differentiation ability of rat white preadipocyte cells using the onion extract and its sulfur-containing components. Cycloalliin, *S*-methyl-L-cysteine, *S*-propyl-L-cysteine sulfoxide, dimethyl trisulfide, especially *S*-methyl-L-cysteine sulfoxide were reported to be effective in inhibiting formation of oil drop in the cells, suggesting that these compounds may be involved in the anti-obesity effect of the onion extract.

## 1. Introduction

Glucose intolerance, marked insulin resistance, and hyperlipidemia were observed in Zucker diabetic fatty (ZDF) (fa/fa) rat. In the prediabetic state, the ZDF rat experiences a steady increase in basal insulinemia and plasma free fatty acid (FFA) levels. Hyperglycemia develops between 8 and 10 weeks of age, leading to overt diabetes and collapsing insulin secretion [1]. This profile mimics the progressive loss of glucose-stimulated insulin secretion in human type 2 diabetes (T2DM) and therefore the ZDF rat represents a good animal model for studying human T2DM pathophysiology and the effects of therapeutic options [2]. 

Onion (*Allium cepa* Linn.) is rich in sulfur-containing compounds and is used as foodstuff, condiment, flavoring and folk medicine. Sulfur-containing compounds in onion may be volatiles and non-volatiles. When cutting onions, the volatile compounds such as dialk(en)yl disulfides and dialk(en)yl trisulfides provide the characteristic flavor and odor through the action of alliinase (E.C. 4.4.1.4, *S*-alk(en)yl-L-cysteine sulfoxide lyase) [3,4]. Non-volatile cysteine sulfoxides (such as *S*-methyl-L-cysteine sulfoxide, *S*-propyl-L-cysteine sulfoxide and *S*-allyl-L-cysteine sulfoxide) are known as the precursors of the volatile compounds. Onion contains a considerable amount of compounds highly beneficial for human health, and has been reported to have antioxidant, anti-hyperglycemic and anti-hyperlipidemic effects on diabetic rats [5,6,7]. Regarding to sulfur-containing compounds, many groups have reported health effects, such as inhibiting human low density lipoprotein oxidation [8]. Kumara *et al.* reported that *S*-methyl-L-cysteine sulfoxide isolated from onion has anti-diabetic, antioxidant and anti-hypolipidemic effects in alloxan diabetic rats [5,9]. Furthermore, it is reported that *S*-methyl-L-cysteine sulfoxide reduced serum and tissue lipid levels in high cholesterol diet fed rats by reducing endogenous lipogenesis, and increasing lipids catabolism and subsequent excretion [10]. However, anti-obesity effect of the sulfur-containing compounds has not yet been reported and there are no studies of the sulfur-containing compounds in onion. Furthermore, the inhibitory effect of sulfur-containing compounds on differentiation of the white adipose cell has not yet been precisely examined.

In the present study, anti-obesity activity of the onion extract was examined on ZDF rats by measuring the efficacy of such relevant markers of diabetes and obesity. In addition, sulfur-containing compounds found in fresh and/or cooked onion were examined to show inhibitory effects on the differentiation of white adipose cell. 

## 2. Materials and Methods

### 2.1. Reagents

Cycloalliin (Cyc) and *S*-methyl-L-cysteine (SMC) were purchased from Wako Pure Chemical Industries, Ltd. (Japan, Osaka). *S*-methyl-L-cysteine sulfoxide (SMCS), *S*-propyl-L-cysteine sulfoxide (SPCS), *S*-allyl-L-cysteine sulfoxide (SACS), dimethyl trisulfide (M2S3), dipropyl trisulfide (P2S3) and diallyl trisulfide (A2S3) were provided by Dr. Hiroyuki Nishimura, professor at Tokai University. 

### 2.2. Animals and Experimental Procedures

Male ZDF (fa/fa) rats (7 weeks old) were purchased from Japan SLC Inc., (Shizuoka, Japan). The rats were maintained with a 12:12 h light-dark cycle at 22 ± 2 °C and 40%–60% humidity. The diet and water were given *ad libitum*. After acclimatizing for 1 week, the ZDF (fa/fa) rats were divided into 4 groups of 6 each, fed on the basal diet (CON group) or basal diet containing onion extract (3% and 5%, w/w) (O3 and O5 groups, respectively) for 28 days. The onion extract was provided from Ryusendo Co., Ltd. (Japan, Tokyo). For onion extract, 10 kg of the fresh onion was heated and then concentrated at 92 °C after squeezing. The volatile constituent that appeared at the time of concentration was absorbed to an active carbon. Ethanol-eluate from the active carbon was evaporated and 1.5 g as the residue was added to the onion extract (1 kg) that was provided for the experiments. The same procedures were adopted with the lean nondiabetic ZDF (+/+) rats (lean group). The composition of each experimental diet can be seen in Table 1. α-Cornstarch: sucrose levels in the onion extract-fed groups were regulated because the onion extract contains more than 90% carbohydrates. Food intake and body weight were determined on daily basis. Blood was collected by cardiac puncture after anesthetizing with Nembutal (Dainippon Pharmaceutical Co., Osaka, Japan) after 10 h of fasting on the last day of the feeding period. Serum was prepared by centrifuging the collected blood at 1000 *g* for 15 min. The liver, left kidney and adipose tissue (mesenteric, left pararenal and epididymal) were detached and weighed. The rats were cared for at all times according to the institutional guidelines of Yamagata University.

**Table 1:** Composition of the diets (%, w/w).

Group	Lean ZDF (+/+)	Diabetic ZDF (fa/fa)		
		CON	O3	O5
Casein	20	20	20	20
*Α*-Cornstarch:sucrose = 2:1	65.5	65.5	62.5	60.5
Corn oil	5	5	5	5
Cellulose	5	5	5	5
Vitamin mixture ^1^	3.5	3.5	3.5	3.5
Mineral mixture ^2^	1	1	1	1
Onion extract	ND	ND	3	5

^1^ AIN-93G-MX and ^2^ AIN-93-VX, which contained 25 g of bitartrate per 100 g, were obtained from Oriental Yeast Co., Ltd. ND means “not added”.

### 2.3. Oral Glucose Tolerance Test (OGTT)

The OGTT was performed on rats that had fasted for 10 h on day 18 of the feeding period in the experimental diet. The rats were administered with a 40% glucose solution (2 g/kg of body weight). Blood was collected from the tail vein 0, 15, 30, 60 and 120 min after glucose loading, and the glucose levels were measured with a Medesafe GR-102 instrument (Terumo Co., Ltd., Tokyo, Japan). The fasting blood glucose level was measured after 10 h of fasting on the last day for the feeding period of the experimental diet. The glucose levels were measured with a Medesafe GR-102.

### 2.4. Serum Insulin and Leptin and Lipid Levels

The levels of serum insulin and leptin were measured with ELISA Levis rat insulin kit (Shibayagi Co., Ltd., Gunma, Japan) and rat leptin ELISA kit (Wako Pure Chemical Industries, Osaka, Japan), respectively. 

Serum total-cholesterol (T-Chol), high density lipoprotein-cholesterol (HDL), triglyceride (TG), and FFA levels were enzymatically measured with commercially available kits conducting cholesterol E test, HDL-cholesterol test, triglyceride E test, and NEFA C test (Wako Pure Chemical Industries, Osaka, Japan). Low density lipoprotein-cholesterol (LDL) level was calculated as: LDL = (T-Chol) − (HDL) − (TG/5) [11].

### 2.5. Cell Culture and Test Sample Treatment

Rat white preadipocyte cells (RWPC) were purchased from TaKaRa Bio Inc. (Tokyo, Japan). They were plated on type Ι collagen-coated dishes and cultured to reach confluence in a standard medium consisting 10% (v/v) of fetal bovine serum, 100 units/mL penicillin, 100 μg/mL streptomycin in Dulbecco’s modified Eagle’s medium (DMEM) at 37 °C in a humidified atmosphere containing 5% CO_2_. When the cells reached confluence, they were incubated in DMEM containing 10 μg/mL insulin, 1 μM dexamethasone, and 0.5 mM 3-isobutyl-1-methylxanthine for 3 days, after which the cells were washed with phosphate-buffered saline (PBS) and incubated in medium containing insulin and varying concentrations of onion extract, the sulfur-containing compound (Cyc, SMC, SMCS, SPCS, SACS, M2S3, P2S3 and A2S3), or the vehicle dimethyl sulfoxide (control group). Seven days after treatment, cells were fixed with 4% formalin in PBS for 30 min at room temperature. After fixation, cells were washed twice with PBS and stained with 0.6% (w/v) Oil Red O in 60% isopropanol for 60 min at room temperature. Cells were washed with water for removal of unbound dye. Dye stained the cells were dissolved with isopropanol and quantitative evaluation of adipogenesis was conducted by measuring absorbance at 540 nm. All experiments with white adipocyte were carried out within two passages.

### 2.6. Cytotoxicity Assay

Cell viability was measured by 3-(4,5-dimethylthiazol-2-yl)-2,5-diphenyltetrazalium bromide (MTT) assay. Before treatment, cells were first left to grow overnight on a 96 well plate at density of 1 × 10^4^ cells/well. After 24 h, onion extract and each sulfur-containing compound were applied to the cells in serum-free DMEM, and cells were incubated for an additional 48 h at 37 °C. The culture medium was aspirated under vacuum and 200 μL MTT (1 mg/mL) was added and further incubated for 4 h at 37 °C. The MTT solution was discarded by aspirating, and the resulting formazan product, which was converted by viable cells, was dissolved in 150 μL dimethylsulfoxide. The absorbance at each well was measured at 540 nm.

### 2.7. Statistics

Each value is given as the mean ± SEM of 6 animals. The homogeneity of variance between treatments was verified by Bartlett’s test. Data were statistically analyzed by a two-way analysis of variance (ANOVA). A *post-hoc* analysis of significance was performed by using Fisher’s PLSD test. Glucose values during the OGTT were analyzed by repeated-measures ANOVA. All comparisons were considered statistically significant at *p* < 0.05. 

## 3. Results and Discussion

In the present study, anti-obesity activity of the onion extract was examined on ZDF rats. Though there was not any difference in food intake and initial body weight among ZDF (fa/fa) groups, body weight gains and adipose tissue weights in the O5 group were found to be lower than the CON group (Table 2). Furthermore, liver weight in onion extract-fed groups exhibited remarkably lower values than the CON group. As serum cholesterol (T-Chol, HDL and LDL) levels were same among ZDF (fa/fa) groups, serum TG and FFA levels in the onion extract-fed groups were found to be significantly lower than the CON group (Table 2). These results are similar to those previously reported [10,12,13].

**Table 2:** Effects of onion extract in the Zucker diabetic fatty (ZDF) rats.

		Lean ZDF (+/+)	Diabetic ZDF (fa/fa)		
			CON	O3	O5
Food intake (g/28 day)		406 ± 12	541 ± 6	540 ± 7	536 ± 11
Initial body weight (g)		187 ± 7	260 ± 6	265 ± 3	257 ± 1
Body weight gain (g/28 day)		65.6 ± 3.2	110.0 ± 5.2 ^a^	106.9 ± 6.6 ^a^	91.6 ± 2.7 ^b^
Food efficiency ^1^		0.162 ± 0.010	0.202 ± 0.011 ^a^	0.195 ± 0.011 ^ab^	0.168 ± 0.005 ^b^
Organ (% of body weight)					
	Liver	2.38 ± 0.30	3.45 ± 0.51 ^a^	3.08 ± 0.23 ^b^	2.76 ± 0.16 ^c^
	Kidney	0.321 ± 0.035	0.235 ± 0.076	0.245 ± 0.057	0.247 ± 0.022
Adipose tissue					
	Mesenteric	0.312 ± 0.012	0.701 ± 0.049 ^a^	0.675 ± 0.087 ^ab^	0.624 ± 0.048 ^b^
	Pararenal	0.691 ± 0.050	1.024 ± 0.061 ^a^	1.033 ± 0.044 ^a^	0.836 ± 0.019 ^b^
	Epididymal	0.606 ± 0.036	1.35.7 ± 0.012 ^a^	1.321 ± 0.073 ^a^	1.090 ± 0.112 ^b^
Fasting blood glucose (mg/dL)		87.8 ± 9.9	152.0 ± 9.3 ^a^	131.7 ± 8.3 ^ab^	117.7 ± 10.1 ^b^
Serum insulin (ng/mL)		1.79 ± 0.35	11.1 ± 0.7	11.3 ± 1.1	10.8 ± 1.3
HOMA-IR ^2^		14.2 ± 3.4	142.7 ± 10.8 ^a^	120.3 ± 14.7 ^ab^	113.2 ± 7.9 ^b^
Serum leptin (ng/mL)		0.214 ± 0.013	14.34 ± 2.01	15.63 ± 1.61	16.30 ± 1.08
Serum lipid					
	T-Chol (mg/dL)	104 ± 4	267 ± 6	258 ± 11	257 ± 6
	HDL (mg/dL)	32.9 ± 1.2	98.6 ± 3.4	84.9 ± 2.6	97.2 ± 2.8
	LDL (mg/dL)	68.1 ± 2.5	130.9 ± 35.2	141.2 ± 11.0	136.8 ± 5.9
	TG (mg/dL)	27.2 ± 8.6	205.5 ± 22.0 ^a^	140.2 ± 17.5 ^b^	113.3 ± 6.4 ^c^
	FFA (mEq/L)	0.663 ± 0.010	1.264 ± 0.118 ^a^	0.922 ± 0.039 ^b^	0.942 ± 0.051 ^b^

Animals were treated for 28 days with the basal diet (lean and CON group) or basal diet containing onion extract (3 and 5%, w/w) (O3 and O5 groups). Values are means ± SEM. ^a, b, c^ Mean values within a row unlike superscript letters were significantly different among ZDF (fa/fa) groups (*p* <0.05). ^1^ Food efficiency = body weight gain/food intake. ^2^ HOMA-IR = (fasting serum insulin × fasting blood glucose)/405.

**Figure 1:**
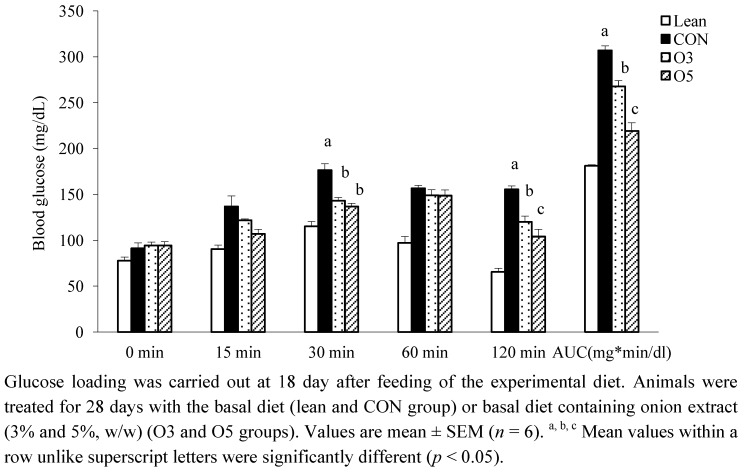
Effects of onion extract on oral glucose tolerance test (OGTT) and the area under the curve (AUC) in the ZDF rats.

The results of OGTT and the area under the curve (AUC) have been shown in Figure 1. After 120 min of glucose loading, blood glucose levels in the onion extract-fed groups were significantly reduced compared to the CON group. The AUC for the onion extract-fed groups was also found to be significantly lower than that of the CON group. Although feeding of onion extract in the O3 group did not report any change in serum levels of fasting blood glucose and HOMA-IR, insulin-resistant index, the levels in the O5 group were significantly lower than those in CON group (Table 2). However, in a later period of the study, the CON group rats were found to be insulin-resistant as compared to those fed with onion extract; no significant increase was observed in the insulin level. These results suggest that dietary onion extract ameliorated glucose tolerance, and then improved insulin resistance. Babu *et al.* have reported that dietary onion ameliorating diabetic nephropathy depends on onion’s ability to lower blood cholesterol levels [14]. Feeding SMCS isolated from onion showed low cholesterol levels in serum and tissues in alloxan diabetic or cholesterol-fed diet rats [9,10]. However, serum cholesterol levels were not different among ZDF (fa/fa) groups in this study. The reason for this phenomenon is not clear, but one reason may be excessive obesity of the ZDF (fa/fa) rat. It has been reported that hyperglycemia is caused by accumulation of the internal adipose tissue [15]. The present results may support the possibility that dietary onion extract improves diabetes by lowering fatty acid levels, not cholesterol. 

**Figure 2:**
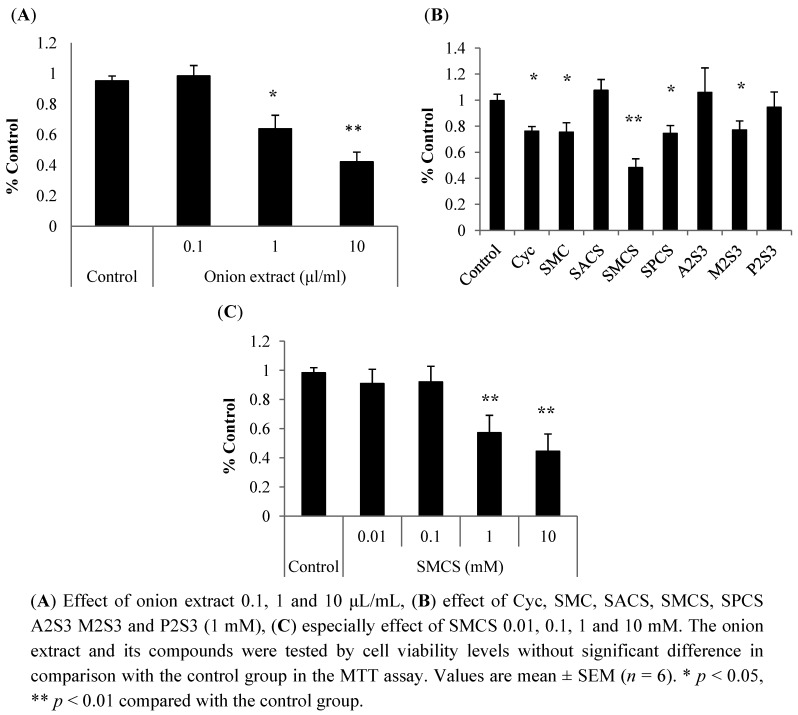
Effects of onion extract and its sulfur-containing compounds on differentiation of Rat white preadipocyte cells (RWPC).

In previous reports, an inhibitory effect on TG and FFA biosynthesis of *S*-allyl cysteine, *S*-ethyl cysteine, SMC, and *S*-propyl cysteine in mice and cultured cell study was observed [12,13,16]. SMCS or SACS are also reported to exhibit anti-diabetic effects [17]. However, anti-obesity effects of the sulfur-containing compounds has not yet been reported. We examined anti-obesity effect of onion extract and its sulfur-containing compounds by using RWPC. At first, onion extract was examined to inhibit the differentiation of RWPC. The differentiations were restrained by density-dependent factor of the onion extract (Figure 2A). When sulfur-containing compounds were examined by this assay, Cyc, SMC, SMCS, SPCS and M2S3 reported strong inhibition, especially SMCS (Figure 2B). These onion extract and sulfur-containing compounds were tested by cell viability levels without significant difference in comparison with the control group in the MTT assay. From these results, compounds having a methyl group were showen to have high inhibitive activity. In addition, SPCS having a propyl group also showed high activity, but SACS which have a double bond did not show any effect. Furthermore, there may be some kind of regularities on chemical structure because SMCS exhibited higher activity than SMC. However, further study is required to understand the reasons for this. The SMCS suppressed the differentiation in a dose-dependent manner, suggesting that the amounts of this compound may also be important in regulating cell differentiation (Figure 2C).

These results suggest that onion extract and its sulfur-containing compounds can suppress lipid accumulation or differentiation in adipocyte. However, it is still to be examined whether the effect of SMCS is observed in the cell experiment on consuming onion or not. Tsuge *et al.* have reported that content of SPCS, the main flavor precursor in onion [18], is more than that of SMCS [19]. Furthermore, Cyc content is increased by the process of heating, and the onion extract reaches about 1 mg/g Cyc. Further evaluation is necessary to examine both the content and the activity.

## 4. Conclusions

In the present study, anti-obesity activity of the onion extract was examined on ZDF rats by measuring obesity and the efficacy of diabetic markers. Serum triglyceride and free fatty acid levels in the onion extract-fed group were found to be significantly lower than the control group without onion extract. Feeding onion extract ameliorated glucose tolerance. Body and adipose tissue weights, fasting blood glucose level were also improved in 5% of onion extract-fed group. These results suggest that dietary onion extract improves diabetes by decreasing triglyceride and free fatty acid levels. The effect of the onion extract seems to be dose-dependent. Furthermore, onion extract and its sulfur-containing compounds were used to examine differentiation ability of rat white preadipocyte cells. Cycloalliin, *S*-methyl-L-cysteine, *S*-propyl-L-cysteine sulfoxide, dimethyl trisulfide, especially *S*-methyl-L-cysteine sulfoxide were found to be effective to inhibit formation of oil drop in the cells, suggesting that these compounds may play a vital role in suppressing obesity. The present study showed that the anti-obesity effect of onion in the rodent that may be beneficial for human health. 
